# Bridging the sensory gap: intraoperative lung ultrasound for deep pulmonary nodule localization in totally endoscopic robotic thoracic surgery

**DOI:** 10.1007/s13304-025-02492-8

**Published:** 2026-01-05

**Authors:** Sebastiano Angelo Bastone, Alexandro Patirelis, Luciano Cialì Sposato, Cristiano Casciani, Karan Kumar, Federico Tacconi, Vincenzo Ambrogi

**Affiliations:** 1 Department of Thoracic Surgery, Tor Vergata University Polyclinic, Viale Oxford, 81, 00133 Rome, Italy; 2 Program in Applied Medical-Surgical Sciences, Department of Surgical Sciences, Tor Vergata University, 00133 Rome, Italy; 3 Doctoral School of Microbiology, Immunology, Infectious Diseases and Transplants, MIMIT, University of Rome “Tor Vergata”, 00133 Rome, Italy

**Keywords:** Intra-operative lung ultrasound (ILU), Robotic assisted thoracic surgery (RATS), Thoracic surgery, Minimally invasive surgery, Pulmonary nodule identification

## Abstract

In robotic-assisted thoracic surgery (RATS), the absence of tactile feedback remains a critical limitation for intra-operative localization of small or deeply located pulmonary nodules. This study aimed to evaluate feasibility and safety of intra-operative lung ultrasound (ILU) as a real-time localization tool in this setting. We prospectively enrolled 26 patients undergoing totally endoscopic RATS for solid or part-solid pulmonary nodules located > 1 cm from the visceral pleura between February 2024 and February 2025. A linear extracorporeal ultrasound probe (Esaote LP 4–13) was used via the assistant port to identify the target lesion. Detection rate, localization time, resection margins, histology at frozen section and intra-operative outcomes were recorded. ILU successfully localized the target nodule in 25 out of 26 patients (96.1%). Median nodule diameter was 12 mm (IQR 9–14 mm), and median localization time was 12 min (IQR 10–15 min). All resected nodules had clear margins, with a median tumor-free distance of 11 mm (IQR 9–13 mm). Lobectomy was completed in 23 cases, while remaining patients underwent wedge resection alone due to indolent histology or intolerance to one-lung ventilation. No conversions to thoracotomy or peri-operative complications were observed. ILU proved to be a safe, accurate, and reproducible method for localizing deep pulmonary nodules in totally endoscopic RATS. By restoring the surgeon’s spatial perception through imaging, ILU effectively compensates for the lack of tactile feedback in robotic surgery. This technique may allow for broader adoption of upfront robotic resections without pre-operative localization or biopsy.

## Introduction

Minimally invasive thoracic surgery has become the cornerstone treatment for early-stage lung cancer and indeterminate pulmonary nodules, with both video-assisted thoracic surgery (VATS) and robotic-assisted thoracic surgery (RATS) being increasingly adopted in clinical practice. Compared to open surgery, these techniques offer reduced post-operative pain, faster recovery, and shorter hospital stays [1, 2]. While RATS provides superior visualization, greater instrument articulation, and improved surgeon ergonomics, it lacks one crucial component, that is, tactile feedback [3, 4]. This limitation represents a significant challenge during robotic resections, particularly for deep or small nodules in a totally endoscopic setting without utility incisions [3].

Several pre-operative localization techniques have been developed to address this issue, including computed tomography (CT)-guided hook-wire or coil placement, methylene blue or radiotracer injection, and navigational bronchoscopy [5–8]. Although effective, these techniques are often invasive, time-consuming, require coordination with interventional radiology, and are associated with complications such as pneumothorax, hemorrhage, or air embolism [5, 6, 9].

Intra-operative lung ultrasound (ILU) has emerged as a non-invasive and real-time alternative. Greenfield et al. firstly described its use in 1997, achieving 92.3% detection of non-palpable nodules during VATS [10]. Kondo et al. later demonstrated its efficacy in detecting ground-glass opacities (GGO) [11]. Subsequent studies confirmed the utility of ILU in identifying sub-centimetric nodules and GGO with high accuracy and safety, often avoiding conversion to thoracotomy or the need for additional resections [9, 12, 13].

Recent comparative data support the superiority of ILU over traditional manual palpation. We have recently reported 100% detection rate and significantly shorter localization and operative times in uniportal VATS with ILU compared to multiportal VATS using manual or instrumental palpation (9 vs. 14 min) [14]. Gambardella et al. found that ILU significantly improved detection of nodules < 10 mm, with a 100% success rate and no procedure-related complications [15].

Despite these promising results in VATS, evidence for ILU in RATS remains still limited. Zhou et al. first described ILU as “an alternative finger” in robotic surgery in 2017 [16]. Fiorelli et al. recently reported a 100% detection rate using ILU during RATS in a small case series, confirming its feasibility [17]. However, no study has systematically assessed its performance in a larger prospective cohort undergoing totally endoscopic RATS for deeply located nodules.

This study aimed to evaluate the feasibility, effectiveness, and safety of ILU for detecting solid or part-solid pulmonary nodules located > 1 cm from the pleural surface during RATS. Primary outcome included detection success rate, while secondary measures focused on localization time, resection margin adequacy and intra- or post-operative complications.

## Material and methods

### Study design

This is a prospective, single-center study conducted from February 2024 to February 2025, with the aim of evaluating the feasibility and accuracy of ILU for detecting pulmonary nodules during totally endoscopic RATS.

The study population included patients referred to our thoracic surgery department for surgical resection of pulmonary nodules with radiological features suspicious of malignancy but no histological diagnosis. Indeed, our hospital is not equipped with navigational bronchoscopy yet. Moreover, it has interventional radiologists able to perform CT-guided biopsies. However, due to their limited number, the high procedural demand from multiple specialties and a lack of a dedicated CT scan for biopsies, they do not have sufficient availability to perform biopsies for all pulmonary nodules. For this reason, we decided to reserve CT-guided biopsies only for pulmonary nodules with indeterminate imaging characteristics. Conversely, nodules with a high suspicion of malignancy are typically referred directly to surgery. Candidates were selected based on the following inclusion criteria: age ≥ 18 years, presence of a solid or part-solid nodule, situated more than 1 cm from the visceral pleural surface on CT scan, and suitability for robotic anatomical lung resection or wedge resection. Patients with pure GGO, known benign lesions, superficially located nodules (≤ 1 cm from visceral pleura), severe emphysema impairing lung deflation, or any contraindications to single-lung ventilation were excluded. Patients with pre-operative diagnosis and with indication to anatomical resection without necessity of wedge for frozen section were also ruled out. Patients requiring conversion to thoracotomy for reasons unrelated to nodule localization were also excluded.

All patients provided written informed consent, and the study was approved by the local Institutional Review Board (approval n 151.24) in accordance with the Declaration of Helsinki.

### Surgical procedure

Surgical procedures were performed using a da Vinci Xi® robotic platform in a completely endoscopic fashion, without the use of a utility incision, by the same surgical team. Particularly, the assistant surgeon who controlled the ultrasound probe had previously attended a specific course in lung ultrasound and had an experience of about 20 cases in ILU during VATS procedures. Patients were placed in lateral decubitus under general anesthesia with selective one-lung ventilation. After port placement and docking, the lung was gently retracted with robotic instruments to expose the area of interest.

**Fig. 1:**
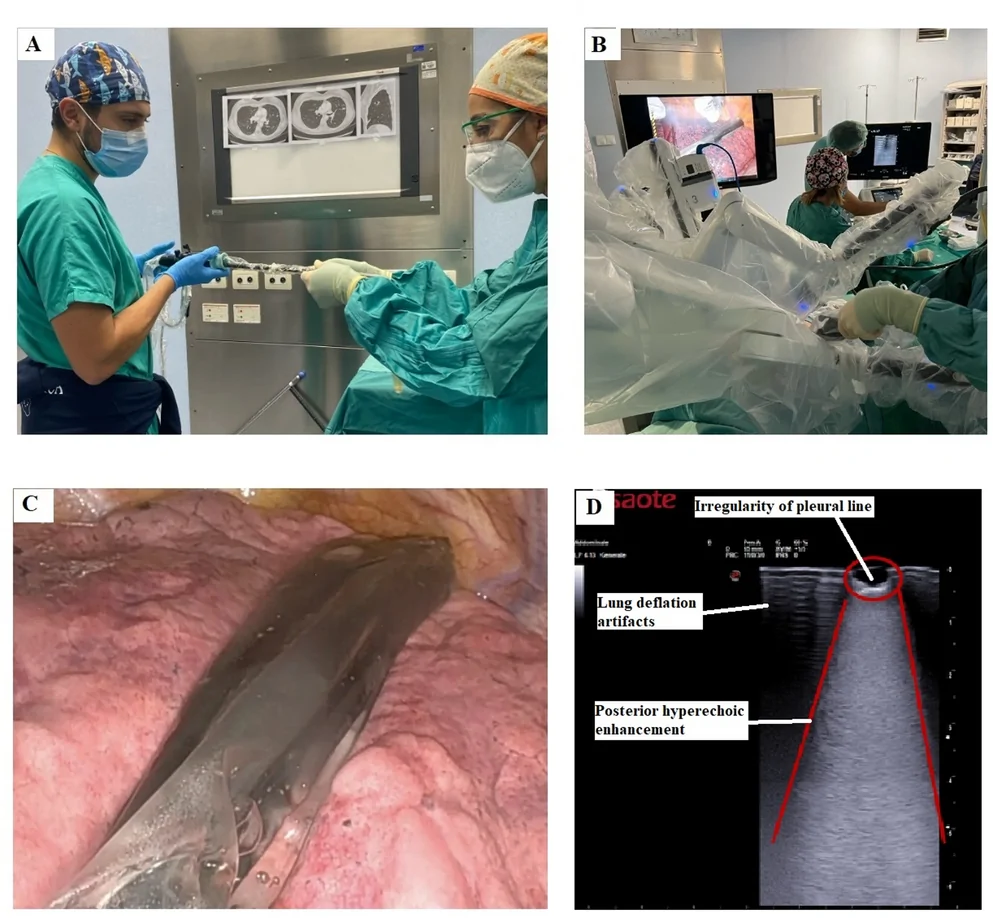
Robotic-assisted thoracic surgery (RATS) with intra-operative lung ultrasound (ILU): preparation (**A**), setting (**B**), probe placed against the collapsed lung surface (**C**), nodule characteristics (**D**)

Nodule localization was performed using a linear ultrasound probe (Esaote LP 4–13®), which measures approximately 43 cm in length and features a 10 cm articulated distal head that allowing 90° left–right and up–down movements. Piezoelectric crystals are distributed on one face in the last 4 cm in length of the probe, with a frequency ranging 13–4 MHz. Based on our experience, the optimal setting for lung nodule detection is an abdominal preset with gain at 70% and a depth between 50 and 60 mm. The probe was external to the robotic system and manually inserted by the assistant surgeon through the about 1 cm assistant port. It was enclosed in a sterile sheath filled with acoustic gel to enhance image resolution and maintain sterility. Once inside the thoracic cavity, the probe was placed directly against the collapsed lung surface, and the area of interest was scanned in real time (Fig. 1).

Lesions were identified based on specific ultrasound characteristics, such as interruption or irregularity of the pleural line, posterior acoustic enhancement or shadowing, and localized hyperechoic or hypoechoic patterns consistent with the CT appearance of the lesion. Sometimes it was also possible to clearly demarcate the margins of the nodule. All these features are quite typical of lung nodule during ILU and allow for a differential diagnosis from atelectasis due to lung deflation where there are no posterior shadowing or hyperechoic enhancement and no margin distinction. After confirming the location, the lung surface was marked using an electrocautery hook, ensuring correspondence with the ultrasound findings.

Wedge resection of the marked area was then performed using robotic staplers. The specimen was retrieved through the assistant port (which was enlarged by approximately 5 mm), manually palpated and then sent for intra-operative frozen section examination. If the lesion was confirmed to be a primary lung cancer, the procedure was completed with a robotic lobectomy, based on pre-operative functional evaluation and intra-operative findings. In cases of benign pathology or metastasis, no further resection was carried out.

### Outcomes and data collection

The primary endpoint of the study was the success rate of intra-operative localization, defined as the ability to correctly identify and resect the target lesion as confirmed by frozen section and final histopathology. Secondary endpoints included localization time (measured from introduction of the probe to marking of the lesion with electrocautery hook), operative time (defined as the time from skin incision to the end of wedge resection for frozen section), tumor-free surgical margin, need for additional resections due to missed nodules and intra- or post-operative complications. Post-operative complications were recorded up to 30 days after surgery.

All clinical and operative data were collected in a dedicated database using SPSS software v26.0 (IBM Corp., Armonk, NY, USA). Continuous variables were reported as median and interquartile range (IQR), while categorical variables were expressed as absolute numbers and percentages. Boxplots were used to graphically represent some variables.

## Results

A total of 26 patients underwent full RATS with ILU for localization of pulmonary nodules between February 2024 and February 2025. Surgery and nodule characteristics are summarized in Table 1.

**Table 1:** Surgery and nodule characteristics of the enrolled population

Variable	
Nodule diameter (mm), median (IQR)	12 (9–14)
Nodule localization, n (%)	
Anterior	15 (57.7%)
Posterior	11 (42.3%)
Interested lobe, n (%)	
Upper lobes	14 (53.8%)
Middle lobe	2 (7.7%)
Lower lobes	10 (38.5%)
Nodule detection, n (%)	
Yes	25 (96.1%)
No	1 (3.9%)
Tumor-free margin (mm), median (IQR)	11 (9–13)
Localization time (min), median (IQR)	12 (10–15)
Operative time (min), median (IQR)	44 (41–50)
Lobectomy performed, n (%)	
Yes	23 (88.4%)
No	3 (11.6%)
Histology, n (%)	
NSCLC	24 (92.3%)
Indolent lesion	2 (7.7%)
Conversion to thoracotomy, n (%)	
Yes	0 (0.0%)
No	26 (100.0%)
Intra-operative complication, n (%)	
Yes	0 (0.0%)
No	26 (100.0%)
30-day post-operative complication, n (%)	
Yes	0 (0.0%)
No	26 (100.0%)

IQR: interquartile range; NSCLC: non-small-cell lung cancer

The median diameter of the nodules was 12 mm (IQR 9–14 mm), and all lesions were located at a distance greater than 1 cm from the visceral pleura on pre-operative CT. Furthermore, most of the nodules were anteriorly located (57.7%) and involved the upper lobes (53.8%). The ILU procedure was successfully completed in 25 out of 26 patients, yielding a localization success rate of 96.1%**.** The only failed case involved a part-solid nodule of approximately 11 mm of total diameter located in segment six of the right lower lobe at a depth of about 1.5 cm from pleural surface at CT imaging, in a patient with mild emphysema.

In all cases with successful localization, the intra-operative ultrasound findings correlated with CT imaging, and the lesion was confirmed at frozen section examination. The median localization time was 12 min (IQR 10–15 min), while median operative time was 44 min (IQR 41–50 min).

A tumor-free resection margin was obtained in all resected lesions, with a median margin distance of 11 mm (IQR 9–13 mm). Lobectomy was then completed in 23 patients following confirmation of non-small cell lung cancer. In the remaining 3 patients, only wedge resection was performed: in one case due to histological confirmation of an indolent lesion (requiring no further treatment), and in another due to intra-operative intolerance to one-lung ventilation. One patient had failed nodule detection with ILU and underwent a second wedge resection based on anatomical landmarks. Final pathology in this case confirmed a benign lesion.

There were no conversions to thoracotomy, and no intra- or 30-day post-operative complications were observed.

## Discussion

Intra-operative localization of pulmonary nodules remains a significant challenge in minimally invasive thoracic surgery, especially in the context of RATS. One of the fundamental limitations of RATS, despite its many advantages in terms of visualization and instrument articulation, is the complete absence of tactile feedback. This lack of haptic sensation makes it particularly difficult to identify non-visible or deeply located lesions, especially when there is no utility incision for digital palpation [3, 4].

Our study demonstrates that ILU can be applied as an effective and safe tool to compensate for the lack of tactile feedback in RATS. Among 26 patients treated with this approach, ILU enabled successful intra-operative localization in 96.1% of cases, with a median localization time of 12 min. In all these cases, the nodule was confirmed by frozen section and the resection margins were oncologically adequate. Notably, no intra- or post-operative complications occurred, reinforcing the safety of the technique.

These findings align with previous evidence from VATS procedures, where the ability to use manual palpation partly mitigates the challenge of identifying small or deep nodules. We have previously reported a 100% detection rate in uniportal VATS using ILU, with significantly shorter detection times than conventional multiportal palpation-based approaches [14]. Gambardella et al. confirmed that ILU improves localization of sub-centimetric nodules (< 10 mm), with no further intra-operative risks or post-operative complications [15]. Messina and Kondo also demonstrated that ILU is effective in identifying GGOs, a class of nodules that are particularly difficult to palpate or visualize [11, 12].

However, in robotic surgery, where the surgeon operates from a console physically separated from the patient, and there is no way to “feel” the lung tissue, the issue becomes more critical. Unlike VATS, where the surgeon may use instruments or fingers through a small incision to probe the parenchyma, RATS offers no tactile interaction whatsoever with the operative field. This limitation increases the risk of mislocalization, incomplete resection, or the need for conversion, particularly in patients with non-visible nodules or challenging anatomies [18].

In this context, ILU provides a surrogate for the surgeon’s hand, offering real-time, high-resolution feedback about the internal structure of the lung. As previously described by Zhou et al., ILU can act as a “surgeon’s alternative finger,” bridging the sensory gap that characterizes robotic surgery [16]. Our study builds on this idea, providing robust, evidence that ILU, used even with a standard extra-robotic linear probe, can deliver this missing information in a safe and effective way. Similarly, Fiorelli et al. demonstrated 100% success with ILU in a smaller robotic cohort, although with longer localization times (median ~ 18.4 min) [17].

An important strength of ILU is that it does not require pre-operative localization techniques, such as CT-guided hook-wire or coil placement, methylene blue injection, or navigational bronchoscopy. These procedures can be logistically complex, costly, and associated with complications such as pneumothorax, bleeding, or air embolism [5–9]. ILU allows for real-time intra-operative identification, enabling more flexible surgical planning, especially valuable in nodules without a prior tissue diagnosis.

In our study, we used a standard linear ultrasound probe introduced via the assistant port. Though not robot-integrated, the probe offered sufficient resolution and maneuverability to enable reliable scanning of the lung parenchyma. This underscores the practicality of ILU even in centers without access to robotic-compatible ultrasound systems, further enhancing its generalizability.

We are perfectly aware that this study has some limitations. Although our sample size is among the largest in the literature for ILU in RATS, it remains relatively small and included over a short time frame. This reduced number limits the possibility of generalizing the results. Furthermore, the technique was performed by surgeons with extensive experience in thoracic ultrasound, which may not reflect the learning curve in other institutions. The study was also restricted to solid and part-solid nodules, excluding pure GGOs, which still present additional challenges in sonographic identification [19]. We also excluded patients with severe emphysema as the presence of artifacts related to air trapping phenomenon could make this tool not suitable for this setting of patients. Lastly, this study does not report any comparison with other localization techniques.

Despite these constraints, our preliminary findings suggest that ILU has the potential to fundamentally enhance RATS, not by replicating traditional methods, but by replacing the missing tactile input with a different, yet equally informative modality. In this sense, ILU serves not merely as an adjunct, but as a sensory extension of the surgeon, offering confidence and precision in situations where vision alone is insufficient.

Looking forward, the development of robot-integrated ultrasound probes, the use of contrast-enhanced ultrasound (CEUS) [20], and potential future integration of autonomous scanning technologies could further expand the role of ILU. Larger multicenter, randomized studies will be essential to validate our findings and to define the optimal role of ultrasound in the future of robotic thoracic surgery.

## Conclusions

ILU represents a valuable and effective tool to assist with the localization of small, deeply located pulmonary nodules during totally endoscopic RATS. In our preliminary experience, ILU allowed rapid and accurate identification of non-visible nodules, providing adequate resection margins without adding further operative risks.

By offering real-time feedback on parenchymal structures, ILU could effectively compensate for the absence of tactile sensation inherent in the robotic platform. This sensory integration would enhance the surgeon’s intra-operative decision-making process and could give its contribution to obtain precise and oncologically safe resections, even when visual hints and pre-operative markings are insufficient or unavailable.

The use of a standard linear ultrasound probe, rather than a robotic-dedicated device, also demonstrates the practical accessibility of this technique across a variety of surgical settings. Our findings suggest that ILU can be readily implemented in RATS workflows, potentially expanding the indications for upfront robotic surgery, including patients without a pre-operative diagnosis or without prior localization procedures.

While larger multicenter and randomized studies are needed to validate these preliminary and still not generalizable results and optimize standardization, possibly comparing this technique with other localization tools, ILU appears to be a safe, reproducible, and valuable adjunct to robotic thoracic surgery—bridging the sensory gap left by the lack of haptic feedback and enabling a more autonomous, image-guided surgical approach.

## Data Availability

All data generated or analysed during this study are included in this published article.
